# On the evolutionary origin of discrete phenotypic plasticity

**DOI:** 10.1093/g3journal/jkae144

**Published:** 2024-07-10

**Authors:** Takahiro Sakamoto, Hideki Innan

**Affiliations:** SOKENDAI, Research Center for Integrative Evolutionary Science, The Graduate University for Advanced Studies, Hayama, Kanagawa 240-0193, Japan; Department of Genomics and Evolutionary Biology, National Institute of Genetics, 1111 Yata, Mishima, Shizuoka 411-8540, Japan; SOKENDAI, Research Center for Integrative Evolutionary Science, The Graduate University for Advanced Studies, Hayama, Kanagawa 240-0193, Japan

**Keywords:** phenotypic plasticity, population genetics, development, gene regulatory network

## Abstract

Phenotypic plasticity provides an attractive strategy for adapting to various environments, but the evolutionary mechanism of the underlying genetic system is poorly understood. We use a simple gene regulatory network model to explore how a species acquires phenotypic plasticity, particularly focusing on discrete phenotypic plasticity, which has been difficult to explain by quantitative genetic models. Our approach employs a population genetic framework that integrates the developmental process, where each individual undergoes growth to develop its phenotype, which subsequently becomes subject to selection pressures. Our model considers two alternative types of environments, with the gene regulatory network including a sensor gene that turns on and off depending on the type of environment. With this assumption, we demonstrate that the system gradually adapts by acquiring the ability to produce two distinct optimum phenotypes under two types of environments without changing genotype, resulting in phenotypic plasticity. We find that the resulting plasticity is often discrete after a lengthy period of evolution. Our results suggest that gene regulatory networks have a notable capacity to flexibly produce various phenotypes in response to environmental changes. This study also shows that the evolutionary dynamics of phenotype may differ significantly between mechanistic-based developmental models and quantitative genetics models, suggesting the utility of incorporating gene regulatory networks into evolutionary models.

## Introduction

**Fig. 1.**
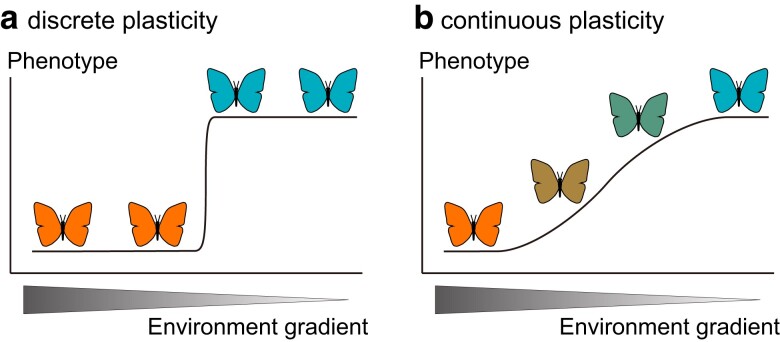
Illustration of discrete and continuous phenotype plasticity.

Phenotypic plasticity is the ability to produce different phenotypes from the same genotype depending on the environment, which can be an attractive strategy in adaptation. Such plasticity should confer a tremendous selective advantage in temporally or spatially fluctuating environments when each phenotype is adaptive in its specific environment. Of special interest is the cases where multiple different phenotypes arise depending on the environment with almost no intermediate phenotypes between them (i.e. discrete phenotypic plasticity), that is, the distribution of phenotype is intrinsically discrete (Fig. 1a). This situation is quite different from continuous plasticity, in which phenotypic trait changes gradually as environment changes (Fig. 1b). There are a number of example of continuous plasticity observed in nature (Huey and Kingsolver 1989; Brakefield and Reitsma 1991). It might be easy to imagine that multiple quantitative loci that express depending on environment could explain continuous phenotypic plasticity, and there are extensive theoretical studies on continuous plasticity, basically using quantitative genetics models (see below). In contrast, discrete phenotypic plasticity seems rare, indicating that discrete phenotypic plasticity is more difficult to evolve than continuous phenotypic plasticity. Examples include temperature-dependent sex determination (Merchant-Larios and Diaz-Hernandez 2013), male horn of beetle (Nijhout 2003), and a mouth form of nematode (Serobyan *et al.* 2014). Discrete phenotypic plasticity should arise through development and require a switch that regulates the expression of many genes concurrently, which should be one of the reasons why discrete phenotypic plasticity hardly evolves. In this study, we present a theoretical model aimed at investigating the evolutionary dynamics of such a system and delineating the conditions under which it could evolve. Our approach employs a population genetic framework that integrates the developmental process, where each individual undergoes growth to develop its phenotype, which subsequently becomes subject to selection pressures. Specifically, we concentrate on elucidating the evolutionary trajectory of the gene regulatory network underlying the developmental process, particularly towards the emergence of discrete phenotypic plasticity.

While this article specifically focuses on discrete plasticity, the majority of previous theoretical studies on phenotypic plasticity have utilized quantitative genetics models, primarily emphasizing continuous plasticity. These models typically assume the presence of numerous loci with small effects that express themselves differentially depending on environmental conditions. Consequently, the collective effects of these quantitative loci give rise to a continuous distribution of phenotypes, making discrete plasticity highly improbable. Within the framework of quantitative genetics models, adaptive plasticity typically evolves in response to either temporally or spatially fluctuating environments (Via and Lande 1985; Gomulkiewicz and Kirkpatrick 1992; Gavrilets and Scheiner 1993). Numerous factors constraining the evolution of plasticity have been extensively explored, including strong genetic constraints (Via and Lande 1985; Gomulkiewicz and Kirkpatrick 1992), costs associated with plasticity (Van Tienderen 1991, 1997), and the overall unpredictability of environmental conditions (Moran 1992; Gavrilets and Scheiner 1993; De Jong 1999; Tufto 2000; Scheiner and Holt 2012). Additionally, some theories have highlighted the potential role of plasticity in rapidly changing environments (Lande 2009; Chevin and Lande 2010; Reed *et al.* 2010; Scheiner *et al.* 2017).

To emphasize discrete plasticity, this study employs a fundamentally different model that integrates the developmental process within each individual. This developmental process is governed by a gene regulatory network, which is believed to play a crucial role in regulating plastic traits in empirical systems (Nijhout 2003; Projecto-Garcia *et al.* 2017; Ledón-Rettig and Ragsdale 2021). In contrast to previous quantitative genetics models, which rely on statistical correlations between phenotype and environments without considering underlying genetic mechanisms (Schlichting and Pigliucci 1993), our model is more mechanistic-based and plausible. By incorporating a gene regulatory network, we aim to capture the mechanistic basis of phenotypic plasticity, reflecting the biological reality of how genes interact to influence phenotypic expression.

We drew inspiration from the work of Watson *et al.* (2014), who demonstrated the “memorizing” capability of gene regulatory networks. In their study, the authors simulated the evolution of a gene regulatory network in alternating environments ($E_{1}\to E_{2}\to E_{1}\to E_{2}\cdots$), each with its specific optimal adult phenotype (i.e. expression pattern). As the network evolved, they gradually adapted to both environments, ultimately acquiring the ability to produce either of the two optimum adult phenotypes based on the genotype of the embryo. This resulted in a discrete distribution of phenotypes, where even slight changes in the embryo genotype (by mutation) could lead to vastly different adult phenotypes. In their model, phenotypic variation is clearly discrete. Here, the network’s input, represented by the embryo expression pattern, functions as a switch that determines the resulting adult phenotype. The reason why this ability can be acquired is because the network has experienced the two environments many times in the past, therefore it evolved such that it can adapt to the two environments with minimum changes in its input.

We consider that such a memory ability of gene regulatory networks should be the mechanism behind discrete phenotypic plasticity. If so, a simple modification of the model of Watson *et al.* (2014) allows to make a realization of phenotypic plasticity with discrete phenotypes. It should be noted that the model of Watson *et al.* (2014) requires mutation to switch the adult genotype. Here, in order to model phenotypic plasticity as defined, our model is designed such that no mutation is needed to switch the adult genotype. To this end, our gene regulatory network model plugs in a sensor gene, whose expression pattern changes depending on the environment. If we consider the sensor gene mimics hormone receptors, this setting should be reasonable according to empirical evidence: It has been reported that hormone receptors play important roles in gene regulatory systems that express only in a sensitive period in development and work as transcription factors when the corresponding hormone exists in that period (Nijhout 2003; Projecto-Garcia *et al.* 2017; Ledón-Rettig and Ragsdale 2021). We demonstrate the successful evolution of a sensor gene into a switch, resulting in the emergence of discrete adaptive phenotypes without alterations in genotype. While our approach may appear straightforward, to our knowledge, there are currently no models that precisely elucidate the mechanisms underlying the evolution of discrete phenotypic plasticity through the modulation of gene regulatory networks (as discussed below).

The evolution of gene regulatory networks has been extensively studied in a wide range of literature. A model of gene regulatory network was first developed by A. Wagner (Wagner 1994, 1996) (see also Kauffman 1969). Since then, it has been widely used to investigate how their structural and functional properties change through evolution in static (Wagner 1996; Siegal and Bergman 2002; Azevedo *et al.* 2006) or fluctuating environments (Crombach and Hogeweg 2008; Draghi and Wagner 2009; Kaneko 2012; Watson *et al.* 2014). Only a few studies have considered phenotypic plasticity by incorporating sensors into the network (Draghi and Whitlock 2012; Iwasaki *et al.* 2013; Brun-Usan *et al.* 2021; Burban *et al.* 2022). These studies showed that the evolution of phenotypic plasticity generates a correlation of phenotypic variation between traits (Draghi and Whitlock 2012; Brun-Usan *et al.* 2021) and affects the amount of phenotypic variation observed among populations that encounter novel environments (Iwasaki *et al.* 2013). Plasticity also affects how gene regulatory networks evolve during domestication processes (Burban *et al.* 2022). However, these studies considered only continuous phenotypic plasticity (but see Iwasaki *et al.* 2013). In addition, previous studies have predominantly focused on the outcomes following the establishment of phenotypic plasticity, rather than on the process of its acquisition. Our work stands out by demonstrating how a network can gradually evolve to acquire discrete plasticity, with a crucial role played by the memory ability of the network. We then explore the conditions necessary for a network to achieve such discrete plasticity.

## Model

We expand upon the model presented by Watson *et al.* (2014) by introducing a sensor gene that functions as an environmental detector. Specifically, we examine two distinct environments, denoted as $E_{1}$ and $E_{2}$, characterized by a single parameter such as temperature. The sensor gene is active only during a sensitive developmental period and its expression level varies depending on the environmental context. For simplicity, we assume that the sensor gene is active in $E_{1}$ and inactive in $E_{2}$. By integrating this sensor gene into the model, our objective is to investigate whether the system can develop phenotypic plasticity, enabling it to produce distinct phenotypes in response to environmental changes, all without altering the genotype. Furthermore, while (Watson *et al.* 2014) employed a deterministic approach, we enhance realism by incorporating stochasticity, specifically random genetic drift, within the context of finite population genetics.

Our model considers a population of haploid species, in which each individual has three types of genes, G, B, and C. There are *n* type-G genes and the phenotype of an individual is defined by their expression pattern, which changes during development. $g_{i}$ represents the genotype of the *i*th type-G gene (i=1,2,3,…,n), which directly determines the expression level of this gene in the embryo ($x_{i}(0)=g_{i}$). $x_{i}$ changes through development, and let $x_{i}(\tau )$ be the expression level of the *i*th type-G gene at the developmental step *τ*. We assume that an embryo becomes an adult after $\tau_{v}$ steps and lives until $\tau =\tau_{l}$. Natural selection works on the expression pattern of the *n* type-G genes, $\vec{x}(\tau )=(x_{1}(\tau ),x_{2}(\tau ),\ldots ,x_{n}(\tau ))$ during adult phase (i.e. $\tau_{v}\leq \tau \leq \tau_{l}$). The types-B and -C genes are involved in this developmental process. $b_{ij}$ determines the regulatory effect of *j*th type-G genes on the expression level of the *i*th type-G genes. Biologically, the type-B genes can be considered as genomic region(s) that consists of regulatory genes/elements of the type-G genes. In addition, the type-C gene determines how the sensor gene affects the expression of the *n* type-G genes ($c_{i}$ denotes the effect on the *i*th type-G gene). Through the developmental process, the expression level of type-G genes changes according to the following formula:


(1) $$x_{i}(\tau +1)=(1-\alpha )x_{i}(\tau )+f(\sum_{\;j=1}^{n}b_{ij}x_{\;j}(\tau )+c_{i}\delta (\tau )),$$

where *α* is the decay rate of gene expression per developmental step. *f* is an activation function: we assume that *f* is a sigmoid function, f(x)=(1+tanh(x))/2, which is a commonly used function to transform any value in (−∞,∞) into the (0,1) interval. In this settings, the minimum and maximum values of $x_{i}$ are 0 and 1/α, respectively. δ(τ) is the expression level of the sensor gene at time step *τ*. In order to make the effect of the sensor gene minimal so that the developmental process mainly depends on the type-B genes, we assume that the sensor gene expresses only in the first $\tau_{c}$ steps of the whole developmental process with $\tau_{l}$ steps (i.e. For $\tau <\tau_{c}$, δ(τ)=1/α in $E_{1}$ and δ(τ)=0 in $E_{2}$). It is important to notice that the absolute expression levels matter only for the type-G genes, on which selection works. The types-B and -C genes determine how the expression level of type-G genes changes over development.

Using this model, we allow all three types of genes to evolve by mutation, selection, and genetic drift, where the environment changes between $E_{1}$ and $E_{2}$ at an interval of $I_{T}$ generations. We then ask if the species can display phenotypic plasticity, which is defined as the ability to develop an adult phenotype which is fit to environment $E_{1}$ if grown in $E_{1}$ and fit to $E_{2}$ when grown in $E_{2}$ from the same genotype (as illustrated in Supplementary Fig. S1). Let ${\vec{X}}_{i}=(X_{i1},\ldots ,X_{in})$ be the optimal expression levels of the *n* type-G genes in environment $E_{i}$ ($X_{ij}\in [0,1/\alpha ]$). The fitness of an adult with $\vec{x}(\tau )$ in environment $E_{i}$ at developmental step *τ* is assumed to be given by $\text{exp}(-|\vec{x}(\tau )-\vec{X_{i}}{|}^{2}/(2\sigma^{2}))$. Then, the lifetime fitness is defined as its geometric mean during the adult phase:


(2) $$\begin{array}{rl} w_{i} & ={\left[\prod_{\tau =\tau_{v}}^{\tau_{l}}\text{exp}(-|\vec{x}(\tau )-{\vec{X}}_{i}{|}^{2}/(2\sigma^{2}))\right]}^{1/(\tau_{l}-\tau_{v}+1)}\\ & =\prod_{\tau =\tau_{v}}^{\tau_{l}}\text{exp}\left(-\frac{\sum_{\;j=1}^{n}[x_{j}(\tau )-X_{ij}{]}^{2}}{2\sigma^{2}(\tau_{l}-\tau_{v}+1)}\right). \end{array}$$

The evolution of the three types of genes proceeds by mutation and selection in a Wright–Fisher haploid population of size *N*. In all individuals, as the initial genotype state, we assume $g_{i}=1/(2\alpha ),b_{ij}=0$, and $c_{i}=0$. Mutation occurs in all three types of genes. $\mu_{g}$ is the mutation rate per type-G gene per individual per generation, which is constant for all *n* type-G genes. A mutation changes $g_{i}$ by Δg, where Δg is a random value drawn from a uniform distribution $U(-\gamma_{g},\gamma_{g})$. We assume $0\leq g_{i}\leq 1/\alpha$, so that $g_{i}=1/\alpha$ if it exceeds 1/α whereas $g_{i}=0$ if $g_{i}$ is smaller than 0. The mutation rate $\mu_{b}$ per generation applies to all $b_{ij}$ of the type-B genes, and each mutation changes $b_{ij}$ by Δb where Δb is drawn from $U(-\gamma_{b},\gamma_{b})$. The mutation rate of each $c_{i}$ of the type-C genes is $\mu_{c}$ per generation, and a mutation changes it by Δc, where Δc is drawn from $U(-\gamma_{c},\gamma_{c})$. At each generation, we compute the fitness of all individuals, and the individuals of the next generation are chosen (free recombination is assumed), to which new mutations are introduced.

Through a simulation run, we compute both $w_{1}$ and $w_{2}$ every generation, where $w_{i}$ is the fitness of the adult individual if it has grown in environment $E_{i}$. The population averages are denoted by ${\bar{w}}_{1}$ and ${\bar{w}}_{2}$, respectively. If the environment is $E_{1}$, only $w_{1}$ is involved in selection and $w_{2}$ is evolutionary meaningless, and vice versa. Nevertheless, it is interesting to monitor both $w_{1}$ and $w_{2}$ through a simulation run to measure the potential ability of the system to fit the two environments.

## Results

### Overview

To investigate the acquisition of phenotypic plasticity through evolution, we conducted comprehensive simulations utilizing the model outlined earlier. Our primary focus is on scenarios with n=8 (referred to as the full model), although we also explore a simpler model featuring two type-G genes (referred to as the simplified model, as detailed below). As the initial state at T=0 for each simulation run in the full model, we set g=(2.5,2.5,2.5,2.5,2.5,2.5,2.5,2.5), c=(0,0,0,0,0,0,0,0), and $b_{ij}=0$, therefore the fitness ($w_{1}$ and $w_{2}$) is relatively low. When both $w_{1}$ and $w_{2}$ increase and become stably close to 1, we consider that phenotypic plasticity is attained. In each run, we simulated at most $10^{7}$ generations and terminated when phenotypic plasticity successfully evolved. We considered that phenotypic plasticity had evolved at generation $T=T_{p}$ if the population mean fitness in both environments (${\bar{w}}_{1}$ and ${\bar{w}}_{2}$) was >0.95 in the following $10^{5}$ generations. If phenotypic plasticity was not acquired until $T=10^{7}$, we considered that plasticity has not evolved successfully. In the following, we consider the parameter values listed in Table 1 unless otherwise specified.

**Table 1.** Default parameter values in the simulation under the full model with eight type-G genes.

Parameters	Explanation	Default
*N*	Population size	1,000
*α*	The decay rate of gene expression	0.2
$\tau_{c}$	The developmental step when the sensor gene expression ends	10
$\tau_{v}$	The developmental step when the expressions start to affect fitness	21
$\tau_{l}$	The developmental step when the expressions end to affect fitness	40
*σ*	Inverse of the strength of selection	5
$\mu_{i}$	Mutation rate per gene for the type-*i* gene	$10^{-5}$
$\gamma_{i}$	Maximum effect size of a new mutation for the type-*i* gene	0.1
$I_{T}$	The number of generations between environmental switch	—
${\vec{X}}_{1}$	Optimal expression pattern in $E_{1}$	(0, 0, 5, 5, 5, 5, 0, 0)
${\vec{X}}_{2}$	Optimal expression pattern in $E_{2}$	(0, 0, 5, 5, 0, 0, 5, 5)

**Fig. 2.**
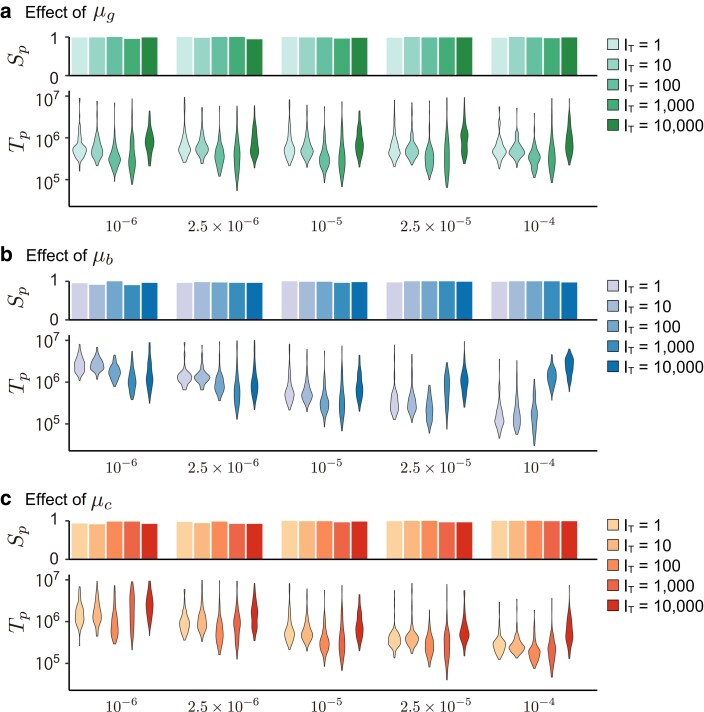
Summary of simulation results in the full model. The effects of the three mutation rates a) $\mu_{g}$, b) $\mu_{b}$, and c) $\mu_{c}$) on the proportion of successful runs ($S_{p}$) and waiting time ($T_{p}$) are shown for various $I_{T}$. In each panel, $S_{p}$ is shown in the upper bar plot while the distribution of $T_{p}$ in successful runs is shown in the lower violin plot. For each $\mu_{i}$, five $\mu_{i}$ values are examined. All other parameters are set to their default values (see Table 1). As a control, the middle column ($\mu_{i}=10^{-5}$) presents the results for the default parameters, so that the results are identical in (a), (b), and (c).

Figure 2 summarizes the simulation results for various pairs of mutation rates and $I_{T}$ under the full model with eight type-G genes. We performed 100 independent simulation runs for each parameter set, from which the proportion of runs that successfully acquired plasticity, $S_{p}$, and $T_{p}$ in the successful runs were recorded. Figure 2 demonstrates that phenotypic plasticity successfully evolves for almost all runs. The waiting time for the acquisition of plasticity ($T_{p}$) tends to be short at intermediate $I_{T}$ ($I_{T}=100,1,000$). It can be explained by the difference of the evolutionary dynamic between small and large $I_{T}$. In the cases of a very small $I_{T}$, the network should perform a challenging task to adapt to both environments almost simultaneously. For a large $I_{T}$, the network can adapt to the environments in turn, perhaps making the adaptation process straightforward. However, there is a drawdown for a large $I_{T}$. When $I_{T}$ is very long, signifying an extended period during which the system adapts to one environment, it becomes challenging to maintain the ability to produce a phenotype suited for the other environment. However, despite this challenge, plasticity eventually evolves after a substantial number of environmental changes, albeit over a prolonged duration. Our findings illustrate that given a sufficiently long time, even with a large $I_{T}$, such as $I_{T}\geq 10^{4}$ generations, the system possesses significant potential to retain past adaptations. This memory capability enables the system to appropriately switch phenotypes based on the prevailing environment.

Although we have provided a brief overview of the simulation results, understanding the impact of each parameter requires examining how the system evolves and ultimately acquires plasticity. To facilitate this understanding, we will first examine the evolutionary process in typical simulation runs. Given the complexity of the dynamics involved in the full model with eight type-G genes, we will begin with a simplified model featuring two type-G genes for elucidation purposes. Subsequently, we will verify that similar dynamics are also observed in the full model. Following a description of the evolutionary mechanism, we will revisit Fig. 2 to discuss the influence of each parameter on the evolution of plasticity.

### Evolutionary dynamics in the simplified model

To understand how the system acquires plasticity, we performed simulations under a very simplified setting with only two type-G genes (n=2). The optimum expression levels of the two type-G genes are ${\vec{X}}_{1}=(5,0)$ in $E_{1}$ and ${\vec{X}}_{2}=(0,5)$ in $E_{2}$. We assumed that a higher mutation rate of $\mu_{i}=10^{-4}$ and stronger selection $\sigma =5/\sqrt{2}$ to account the reduced number of genes compared with the full model. All other parameters are same as those in Table 1.

**Fig. 3.**
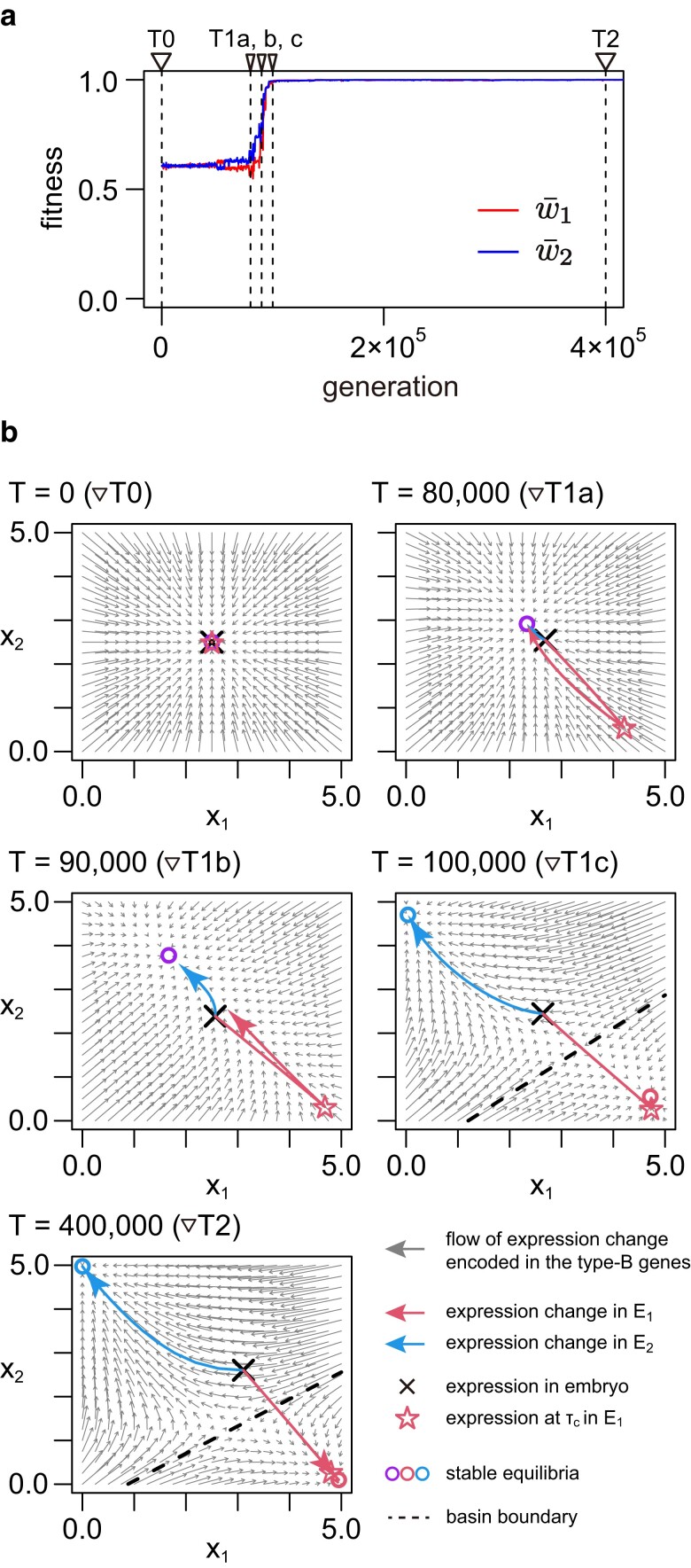
Evolution of phenotypic plasticity in the simplified model with two type-G genes when $I_{T}=1$. The result of a single representative run is illustrated. a) Temporal change of fitness through evolution. The red and blue lines are the fitness in environment $E_{1}$ and that in $E_{2}$, respectively (i.e. ${\bar{w}}_{1}$ and ${\bar{w}}_{2}$). b) Phase portraits of the development of a single individual at five time points (T=0,80,000,90,000,100,000, and 400,000) shown by the dashed vertical lines in (a). ($x_{1}(\tau )$, $x_{2}(\tau )$) starts at × at embryo and moves along the red and blue arrows in $E_{1}$ and $E_{2}$, respectively. The star represents ($x_{1}(\tau_{c})$, $x_{2}(\tau_{c})$) when the activation of the switch ends in $E_{1}$. The default parameter values were used (see Table 1) except for $\mu_{i}=10^{-4}$, $\sigma =5/\sqrt{2}$, ${\vec{X}}_{1}=(5,0)$, and ${\vec{X}}_{2}=(0,5)$.

**Fig. 4.**
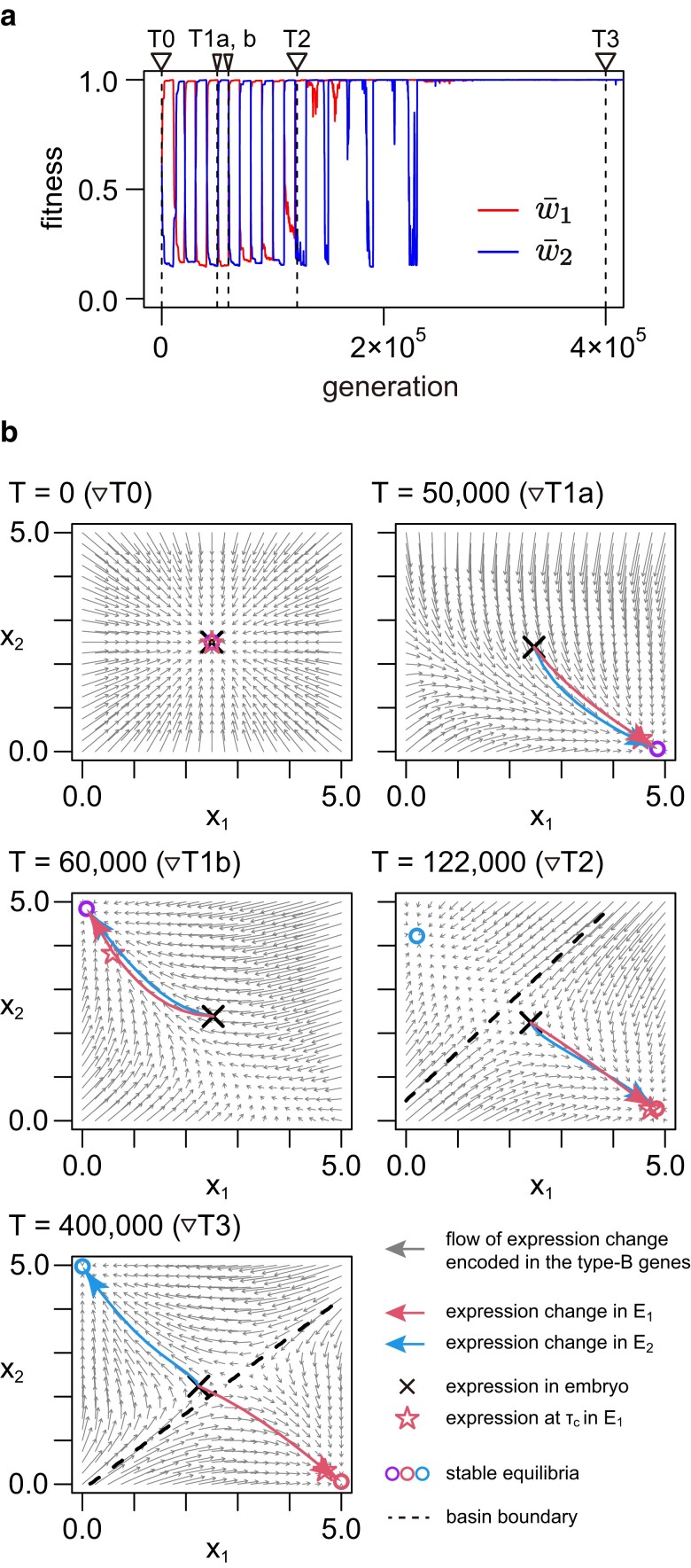
Evolution of phenotypic plasticity in the simplified model with two type-G genes when $I_{T}=10,000$. The result of a single representative run is illustrated. a) Temporal change of fitness through evolution. The red and blue lines are the fitness in environment $E_{1}$ and that in $E_{2}$, respectively (i.e. ${\bar{w}}_{1}$ and ${\bar{w}}_{2}$). b) Phase portraits of the development of a single individual at five time points (T=0,50,000,60,000,122,000, and 400,000) shown by the dashed vertical lines in (a). ($x_{1}(\tau )$, $x_{2}(\tau )$) starts at × at embryo, and moves along the red and blue arrows in $E_{1}$ and $E_{2}$, respectively. The star represents ($x_{1}(\tau_{c})$, $x_{2}(\tau_{c})$) when the activation of the switch ends in E1. The default parameter values were used (see Table 1) except for $\mu_{i}=10^{-4}$, $\sigma =5/\sqrt{2}$, ${\vec{X}}_{1}=(5,0)$, and ${\vec{X}}_{2}=(0,5)$.

Figures 3 and 4 detail typical behaviors of the evolutionary process towards plasticity. The process is quantitatively quite different when $I_{T}$ is very small and when $I_{T}$ is very large, therefore we consider two cases, $I_{T}=1$ and 10,000. The adaptation process to multiple environments in each run can be easily understood by examining the trajectories of the two fitness values, ${\bar{w}}_{1}$ and ${\bar{w}}_{2}$. We first consider the case of $I_{T}=1$, where the environment changes every generation and the system has to adapt to both environments almost simultaneously. Figure 3 shows the evolutionary trajectory in a represented run, in which, after ${\bar{w}}_{1}$ and ${\bar{w}}_{2}$ stay for a while at ${\bar{w}}_{1}∼{\bar{w}}_{2}∼0.6$, they jump up and reach a plateau at ${\bar{w}}_{1}∼{\bar{w}}_{2}∼1$ (see Fig. 3a).

Figure 3b shows how the expression levels of the two type-G genes ($x_{1}$ and $x_{2}$) changes along development in each individual at five time points. At T=0, we set g=(2.5,2.5), c=(0,0), and $b_{ij}=0$, therefore at embryo, ($x_{1}(0)$, $x_{2}(0)$) locates at the center (2.5, 2.5). As this is a stable equilibrium in the developmental dynamics defined by the type-B genes ($b_{ij}$), ($x_{1}(\tau )$, $x_{2}(\tau )$) does not change along development, resulting in ($x_{1}(\tau )$, $x_{2}(\tau )$)=(2.5, 2.5) for any *τ*.

At T=80,000, with ${\bar{w}}_{1}∼{\bar{w}}_{2}∼0.6$ (time point ▽T1a in Fig. 3), both ${\bar{w}}_{1}$ and ${\bar{w}}_{2}$ begin to increase as $b_{ij}$, $c_{i}$, and $g_{i}$ evolve, bringing the adult phenotype closer to its optimum in both environments. At this stage, there is only one stable equilibrium close to the center in the phase portrait (depicted by the purple circle), indicating that individuals develop towards this equilibrium regardless of the environment. However, the difference between the two environments lies in the developmental trajectory: in $E_{2}$, ($x_{1}(\tau )$, $x_{2}(\tau )$) moves directly towards the purple circle (as indicated by the blue arrow), while in $E_{1}$, the evolved switch causes ($x_{1}(\tau )$, $x_{2}(\tau )$) to initially jump to the star before eventually reaching the equilibrium. A similar pattern is observed at T=90,000 (time point ▽ T1b), except that the limited number of developmental steps prevents ($x_{1}(\tau_{l})$, $x_{2}(\tau_{l})$) from reaching the equilibrium. During this phase, the network exhibits only one equilibrium, indicating a phenotype that is moderately adapted to both environments. It appears that the network is still struggling with how to fully adapt to the alternating environments.

A drastic change arises at T=100,000 (time point ▽ T1c), where both ${\bar{w}}_{1}$ and ${\bar{w}}_{2}$ are close to 1. This means that plasticity is almost achieved, and the reason why a single genotype can design two distinct phenotypes is as follows. The key is that the network has evolved such that Equation (1) defined by the type-B genes has two stable equilibria, one close to the top-left corner (blue circle) and the other is the bottom-right corner (red circle), which are obviously the two optimum phenotypes in the two environments. A dashed line is introduced such that ($x_{1}(\tau )$, $x_{2}(\tau )$) transitions towards the blue circle when above the line and towards the red circle when below it (i.e. basin boundary). Consequently, if an individual develops in $E_{2}$, ($x_{1}(\tau )$, $x_{2}(\tau )$) directly converges to the equilibrium near the optimal blue circle. Conversely, in $E_{1}$, where the switch is activated, ($x_{1}(\tau )$, $x_{2}(\tau )$) initially crosses the line towards the star, subsequently transitioning automatically to the red circle. In this scenario, plasticity is deemed to be nearly acquired. As the system evolves further, the two equilibria converge near (0,5) and (5,0), where both ${\bar{w}}_{1}$ and ${\bar{w}}_{2}$ approach 1 (T=400,000). While the expression levels of the embryo ($x_{1}(0)$, $x_{2}(0)$) have shifted slightly from the center, their contribution to the evolution of plasticity diminishes.

Figure 4 demonstrates how phenotypic plasticity evolves with a very large $I_{T}$. Starting at the same initial state as in Fig. 3, the system immediately reaches a phase where ${\bar{w}}_{1}$ and ${\bar{w}}_{2}$ oscillate dramatically (Fig. 4a). For instance, in $E_{1}$ at time point ▽ T1a, the equilibrium approaches (5,0), indicating adaptation solely to $E_{1}$. Consequently, the developmental outcome is uniform across environments, with development consistently progressing to the single equilibrium (purple circle). After an environment change, the system tries to adapt to $E_{2}$ and the equilibrium moves to (0,5) at time point ▽ T1b.

Repeated adaptation to the two environments leads to a qualitative change in the phase portrait. For instance, consider time point ▽ T2, at which the system is in $E_{1}$. Although the developmental outcome remains consistent across environments, we observe an additional equilibrium near the optimal expression for $E_{2}$ (i.e. the top-left corner). This new equilibrium is a consequence of the system’s prior adaptation to $E_{2}$, which persists even in the current phase within $E_{1}$, albeit unused. This scenario echoes the concept of “memorization” of past adaptations as described by Watson *et al.* (2014). By time point ▽ T3, the network has acquired phenotypic plasticity through the successful evolution of the switch function of the type-C gene. This allows ($x_{1}(\tau )$, $x_{2}(\tau )$) to traverse the dashed line when the switch is activated. Consequently, the two equilibria are well-adapted, situated very close to (0,5) and (5,0).

Thus, our simulation under the simplified model effectively demonstrates the fundamental mechanism underlying the acquisition of discrete phenotypic plasticity. Two key conditions appear necessary for achieving phenotypic plasticity: (1) The gene regulatory network must possess a memory of past adaptations, manifesting as two stable equilibria corresponding to the two optimal expression patterns. (2) The type-C gene must facilitate the traversal of the developmental phenotype (expression) across the boundary of the basin during development. With these conditions in place, our model inherently generates a system in which only two distinct phenotypes emerge. However, there may be exceptions, such as instances where some individuals fail to reach an equilibrium in the developmental process due to slow convergence. In these cases, intermediate phenotypes may be observed, potentially leading to a nondiscrete distribution of phenotypes.

It would be intriguing to explore the scenario where an evolved individual with acquired plasticity is exposed to an intermediate environment between $E_{1}$ and $E_{2}$. In such a situation, we would expect the switch to be partially activated. Nevertheless, the system would still produce two phenotypes as long as the developmental process occurs rapidly enough for all individuals to develop optimal phenotypes. Intermediate phenotypes could arise if the partially activated switch impedes ($x_{1}(\tau )$, $x_{2}(\tau )$) from effectively crossing the basin boundary (i.e. if the point represented by the star is located near the boundary), preventing it from reaching the optimum before reaching adulthood. To explore these possibilities further, we will return to the full model with eight type-G genes to consider more realistic scenarios.

### Cases of fast environment transition in the full model

**Fig. 5.**
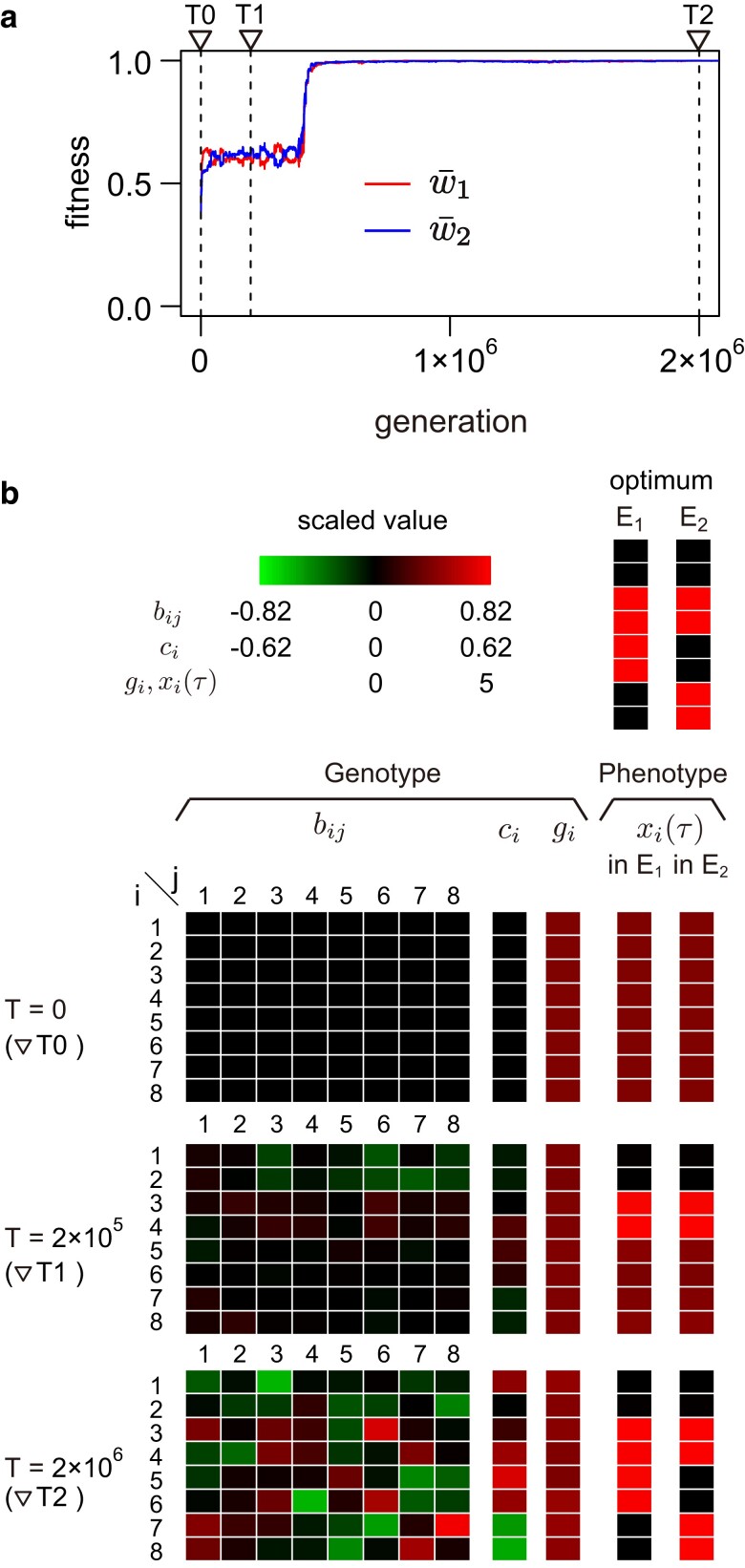
Evolution of phenotypic plasticity in the full model when $I_{T}=1$. The result of a single representative run is illustrated. a) Temporal change of fitness through evolution. The red and blue lines are the fitness in environment $E_{1}$ and that in $E_{2}$, respectively (i.e. ${\bar{w}}_{1}$ and ${\bar{w}}_{2}$). b) Genotype and phenotype of an adult in each environment at three time points ($T=0,2\times 10^{5}$, and $2\times 10^{6}$) shown by the dashed vertical lines in (a). Phenotypes at the middle of the adult phase τ=30 are shown. The default parameter values were used (see Table 1).

Following Figs. 3 and 4, we trace the adaptation process to multiple environments in each run by examining the trajectories of the two fitness values, ${\bar{w}}_{1}$ and ${\bar{w}}_{2}$. We first consider the case of $I_{T}=1$, where the environment changes every generation and the system has to adapt to both environments almost simultaneously. The overall pattern is very similar to that shown in Fig. 3. Figure 5 shows the evolutionary trajectory in a represented run, where both ${\bar{w}}_{1}$ and ${\bar{w}}_{2}$ increase simultaneously in the earlier phases, and after staying for a while at ${\bar{w}}_{1}∼{\bar{w}}_{2}∼0.6$ they increase and achieve ${\bar{w}}_{1}∼{\bar{w}}_{2}∼1$ (see Fig. 5a). Since it is no longer feasible to make a phase portrait in the full model, we below see the evolutionary dynamics focusing on the genotype and adult phenotype.

In this run, at T=0, we set g=(2.5,2.5,2.5,2.5,2.5,2.5,2.5,2.5), c=(0,0,0,0,0,0,0,0) and $b_{ij}=0$. With this setting, the adult phenotype at T=0 is $\vec{x}(\tau )=g$ whichever environment it is grown in (all genes are in brown in Fig. 5b), which are quite different from the two optimum phenotypes so that the fitness is quite low (${\bar{w}}_{1}∼{\bar{w}}_{2}∼0.3$) (time point ▽ T0 in Fig. 5). ${\bar{w}}_{1}$ and ${\bar{w}}_{2}$ then start increasing along the evolution of $b_{ij}$, $c_{i}$ and $g_{i}$ to make adult phenotype close to its optimum in both environments. At $T=2\times 10^{5}$ with ${\bar{w}}_{1}∼{\bar{w}}_{2}∼0.6$ (time point ▽T1 in Fig. 5), the expression patterns of the first four type-G genes (genes 1, 2, 3, and 4) are already very close to the optimums, which is easy to understand because their optimum expression patterns are shared by the two environments. In other words, the evolution of these genes are rather straightforward with a single goal (i.e. optimum expression), which does not apply to the other four genes with two distinct goals. At time point ▽T1, genes 5, 6, 7, and 8 have not adapted to either of the two environments, exhibiting similar adult expression levels in the two environments. In other words, the network can produce only one kind of phenotype that are moderately adapted to the two environments, consistent with time point ▽ T1a in Fig. 3.

The network eventually acquires the ability to produce two optimum phenotypes almost completely around time $T=4\times 10^{5}$ (see Fig. 5a). At time $T=2\times 10^{6}$ (time point ▽T2 in Fig. 5), genes 5, 6, 7, and 8 have distinct expression levels depending on the environment, resulting in two distinct phenotypes. The sensor gene (type-C gene) has evolved as an initial trigger for developmental plasticity by enhancing type-G genes 5 and 6 and suppressing type-G genes 7 and 8 in $E_{1}$. In $E_{2}$, high expression of type-G genes 7 and 8 and very low expression of type-G genes 5 and 6 are produced without the expression of the sensor gene (directly through the regulation network of type-B genes itself). The relative contribution of type-G genes to the acquisition of plasticity seems low.

Similar patterns were observed in almost all runs (see Supplementary Fig. S2 for other runs). It can be summarized that the adaptation process generally involves two steps when $I_{T}$ is small. In the first step, the network evolves such that it produces a single kind of phenotype (whichever the sensor gene is active or inactive) that is equally fit to the two environments, but the fitness is fairly low. Then, the network struggles for a while to evolve to be able to produce two optimum phenotypes. During this process, we do not observe a marked increase in fitness because fitting to one environment likely has a negative effect on fitting to the other environment. In the meantime, the network still keeps evolving, and at some point, it becomes able to produce two optimum phenotypes when both ${\bar{w}}_{1}$ and ${\bar{w}}_{2}$ dramatically increase to almost 1 (second step). We can consider that, at this stage, the network has “memorized” the two environments, obtaining the ability to produce two adaptive phenotypes with a help of the switch function by the sensor gene.

### Cases of slow environment transition in the full model

**Fig. 6.**
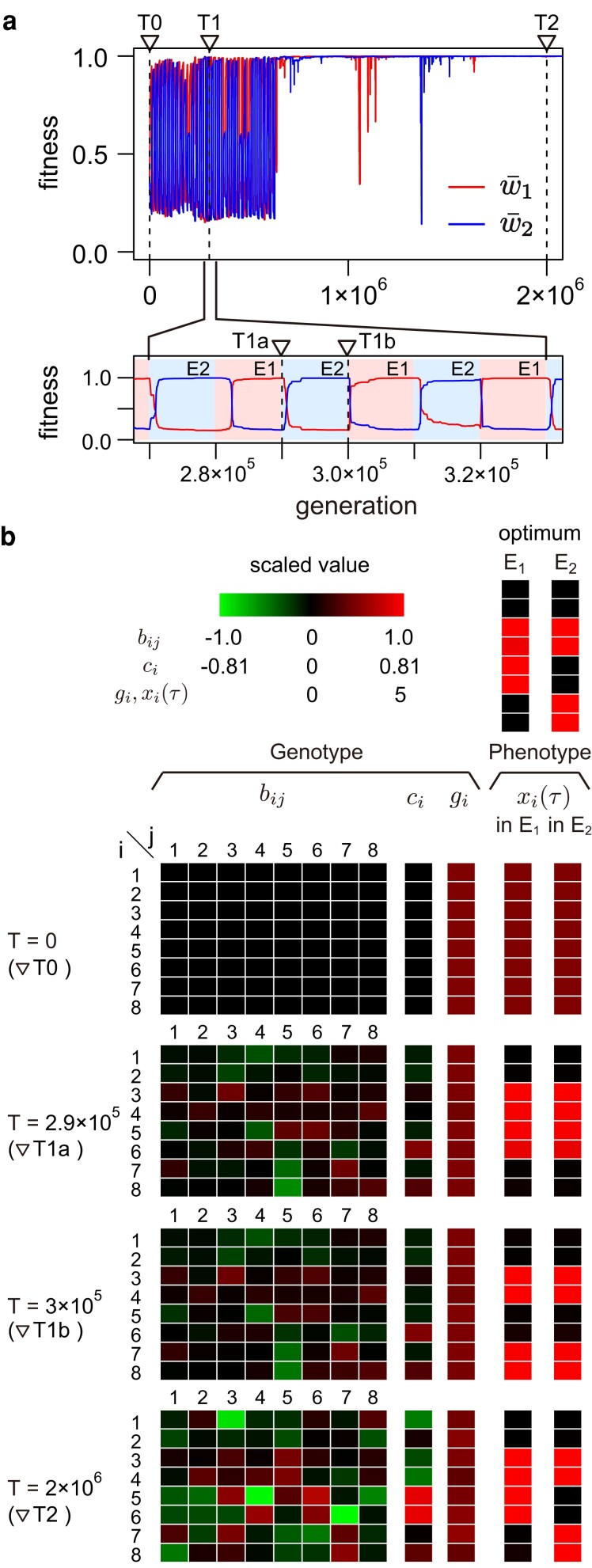
Evolution of phenotypic plasticity in the full model when $I_{T}=10,000$. The result of a single representative run is illustrated. a) Temporal change of fitness through evolution. The red and blue lines are the fitness in environment $E_{1}$ and that in $E_{2}$, respectively (i.e. ${\bar{w}}_{1}$ and ${\bar{w}}_{2}$). b) Genotype and phenotype of an adult in each environment at four time points ($T=0,2.9\times 10^{5},3\times 10^{5}$, and $2\times 10^{6}$) shown by the three dashed vertical lines in (a). Phenotypes at the middle of the adult phase τ=30 are shown. The default parameter values were used (see Table 1).

Next, we focus on how phenotypic plasticity evolves with a very large $I_{T}$. Figure 6 shows the result of a representative run when $I_{T}$ is very large ($I_{T}=10,000$). The pattern is quite different from that for a small $I_{T}$ in the previous section. Starting at the same initial state as in Fig. 5, the system immediately reaches a phase where ${\bar{w}}_{1}$ and ${\bar{w}}_{2}$ dramatically fluctuate (Fig. 6). In the earlier stages in this phase, the fitness increases gradually after each environment change, and then the fitness’ recovery becomes faster over time. As is clearly seen in the close-up of Fig. 6a, after an environment change, for instance from $E_{1}$ to $E_{2}$ (e.g. at time point ▽ T1a), ${\bar{w}}_{2}$ suddenly increases to ∼1 whereas ${\bar{w}}_{1}$ decreases from ∼1 (i.e. losing the ability to adapt to $E_{1}$), and vice versa (e.g. at time point ▽ T1b). The observed immediate adaptation suggests that the system has evolved the ability to adapt to the environment change very quickly through a few mutations after experiencing adaptation to the same environment, consistent with the result of Crombach and Hogeweg (2008). This means that, during this phase with fluctuating ${\bar{w}}_{1}$ and ${\bar{w}}_{2}$, the network gradually “memorizes” how to adapt to each of the two environments. At the moment, the network can produce two phenotypes by a minimum number of mutations, but the sensor gene does not work well as a switch (i.e. phenotypic plasticity has not evolved yet).

The middle two panels in Fig. 6b show the expression patterns at time points ▽ T1a and ▽ T1b, where the average of ${\bar{w}}_{1}$ and ${\bar{w}}_{2}$ is roughly 0.6. At time point ▽ T1a (the end of $E_{1}$), the system is fully adapted to $E_{1}$, but not yet to $E_{2}$. The expression patterns of the type-G genes in $E_{1}$ and $E_{2}$ are very similar to each other and fit to only $E_{1}$. After $I_{T}=10,000$ generations at time point ▽ T1b (the end of $E_{2}$), the expression patterns of the type-G genes in $E_{1}$ and $E_{2}$ are again very similar to each other and fit to only $E_{2}$. This phase is comparable with time points ▽ T1a and ▽ T1b in the simplified model (Fig. 4). It is interesting to note that the clear difference in the expression patterns of type-G genes between ▽T1a and ▽T1b is caused by a very little change in $b_{ij}$, $c_{i}$, and $g_{i}$, and no notable difference is observed. The network still needs to evolve such that it can switch the two phenotypes even more easily, ideally by the sensor gene alone (without changing the network itself by mutation). The phase of alternate fluctuation of ${\bar{w}}_{1}$ and ${\bar{w}}_{2}$ continues for quite long, then the system suddenly acquires the ability to fit to both environments (${\bar{w}}_{1}∼{\bar{w}}_{2}∼1$) around $T=8\times 10^{5}$. Even after this event, we can observe a drastic drop of fitness, but it recovers very quickly.

The bottom panel of Fig. 6b shows the expression patterns at time point ▽ T2, where the system successfully has adapted to the two distinct optimums ${\vec{X}}_{i}$ without changing $b_{ij}$, $c_{i}$ and $g_{i}$. The types-B and -C genes have contributed to this acquisition of plasticity, which is seen in well activated $b_{ij}$ and $c_{i}$ for i,j≥5. The relative contribution of type-G genes to the acquisition of plasticity seems low.

This typical pattern is observed in almost all runs using this parameter set (see Supplementary Fig. S3). The pattern is quite different from the case of a small $I_{T}$ where the system attempts to adapt to the two environments simultaneously, indicating $I_{T}$ affects how a network acquires plasticity. It seems that, while repeatedly adapting to one of the two environments, the network gradually “memorizes” how to adapt, and becomes possible to adapt to environmental changes with minimum modifications by mutation. Then, the sensor gene eventually evolves as an effective switch, which offers an ability to adapt the two environment with no mutational changes in the system.

Thus far, we showed two typical patterns for small and large $I_{T}$. We found that, in cases with intermediate $I_{T}$, the pattern is a mixture of the two extreme cases. See Supplementary SI for details.

### Effect of mutation parameters

Based on the detailed understanding of the evolutionary dynamics toward discrete plasticity (see above), we explore the effect of mutation parameters in the full model with eight genes. In Fig. 2, we summarize how each of the three mutation rates ($\mu_{g}$, $\mu_{b}$, $\mu_{c}$) affects $S_{p}$ and $T_{p}$. In general, they have small effects on $S_{p}$, while $T_{p}$ is quite much affected by the mutation rates. $\mu_{c}$ is the mutation rate at the switch gene. Since quick changes of the switch gene enhances the ability to react environmental changes and promote the acquisition of plasticity, $T_{p}$ decreases as $\mu_{c}$ increases, irrespective of $I_{T}$. The mutation rate of the type-B genes ($\mu_{b}$) has a more complicated effect: $\mu_{b}$ decreases $T_{p}$ when $I_{T}$ is small, while it slightly increases $T_{p}$ when $I_{T}$ is large. This pattern is explained by the difference in the evolutionary dynamics between the small and the large $I_{T}$ cases (see Figs. 5 and 6). When $I_{T}$ is small, the network needs to adapt to the two environments almost simultaneously, which requires joint evolution of the type-B and the type-C genes. Since the faster evolution of the type-B genes makes the acquisition of this ability easier (as well as type-C-gene), $\mu_{b}$ has a negative correlation with $T_{p}$. In contrast, when $I_{T}$ is large, the gene regulatory network adapts to each environment one by one. This process can be rephrased as follows. When the network tries to adapt to one environment, it has to modify the network that was optimized to the other environment. That is, learning (adapting to) one environment is coupled with reducing the fitness to the other (i.e. forgetting the past adaptation). Mutations in the type-B genes not only promotes adaptation to the current environment but also losing adaptation to the previous environment. Therefore, when $I_{T}$ is large, $\mu_{b}$ has a slightly positive correlation with $T_{p}$. The effect of $\mu_{g}$ seems small, suggesting that $g_{i}$ is relatively less important in the evolution of plasticity.

We also examined the effect of mutational impacts ($\gamma_{g}$, $\gamma_{b}$, $\gamma_{c}$) while assuming $\mu_{g}=\mu_{b}=\mu_{c}=10^{-5}$ (Supplementary Fig. S4). The basic pattern of the effect of $\gamma_{i}$ is very similar to that of $\mu_{i}$, although changes in $\gamma_{i}$ have a greater impact on $T_{p}$ and also have some impact on $S_{p}$. As $\gamma_{c}$ increases, plasticity is more likely to evolve in a short time. A large $\gamma_{b}$ tends to decrease $T_{p}$ when $I_{T}$ is small, while increases $T_{p}$ when $I_{T}$ is large. $S_{p}$ becomes small in the parameter space where $T_{p}$ is very long (i.e. small $\gamma_{c}$, and large $\gamma_{b}$ in large $I_{T}$). No clear effect of $\gamma_{g}$ is observed, consistent with the absence of the effect of $\mu_{g}$.

### Discreteness of phenotype

Our model thus far considered two distinct environments, $E_{1}$ and $E_{2}$, each of which has its optimum phenotype, and a successful adaptation to both environments is required to evolve plasticity with discrete phenotypes. It was assumed that the sensor gene is active in $E_{1}$ and inactive in $E_{2}$, or the expression level is 1/α and 0 in $E_{1}$ and $E_{2}$, respectively. To check whether the evolved plasticity is discrete or continuous, it would be intriguing what kind of phenotype arises if the environment is somewhere between $E_{1}$ and $E_{2}$. We here consider intermediate environments between $E_{1}$ and $E_{2}$, which can be realized by assuming intermediate levels of expression of the sensor gene between 0 and 1/α.

**Fig. 7.**
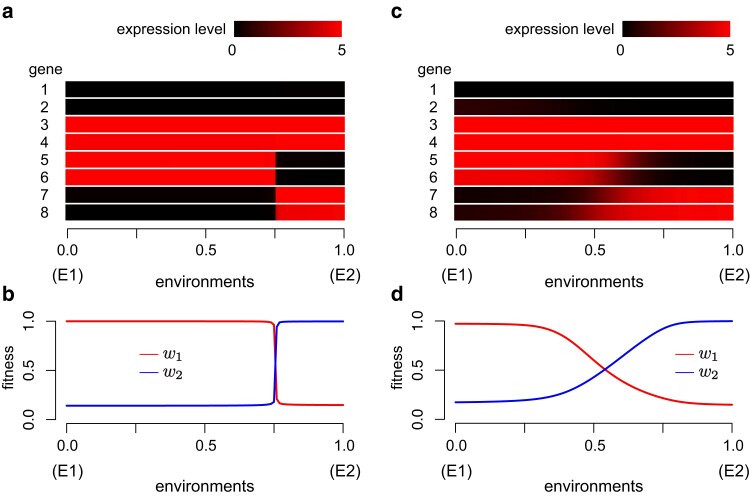
Discreteness of phenotype distribution in intermediate environment. a, b) An example case with a highly discrete distribution. Expression patterns of the 8 type-G genes a) and fitness values in $E_{1}$ and $E_{2}$ b) are plotted. Default parameters and $I_{T}=1$ were assumed. c, d) An example case with a less discrete distribution. Default parameters except for $\mu_{c}=10^{-6}$ and $I_{T}=1$ were assumed.

With this simple assumption, we re-examined the simulation results. Each simulation run produced an evolved gene regulatory network (types-G, B, C genes), which can be used to reproduce adult phenotypes under any environment. In practice, after each run, for each individual, a large number of its embryos were grown in intermediate environments. We then judged that the distribution is discrete if only two optimum phenotypes arise in almost all range of the environment. Figure 7a shows a typical example of an individual with discrete plasticity. Focus on the expression patterns of genes 5, 6, 7, and 8 that distinguish the two optimum phenotypes, ${\vec{X}}_{1}$ and ${\vec{X}}_{2}$. When the environment is changed from 0 to 1 (i.e. from $E_{1}$ to $E_{2}$), we observe that the expression levels of these genes abruptly change between 0 and 5 around when the environmental parameter is 0.75, indicating that we can consider that only two types of phenotypes arise in almost all range of the interval. This pattern is also well-characterized by the distribution of $w_{1}$ and $w_{2}$: When the distribution is discrete, in almost all range, either $w_{1}$ or $w_{2}$ is nearly 1, with only a small range where both $w_{1}$ are $w_{2}$ are significantly smaller than 1. In Fig. 7c, a typical pattern of the cases of continuous phenotypes is shown. When the environment is transitioned from 0 to 1, the expression levels of genes 5, 6, 7, and 8 change gradually. As a consequence, $w_{1}$ and $w_{2}$ change gradually and there is a fairly large range of the environment that produces “intermediate phenotypes” (i.e. phenotypes that does not fit to either $E_{1}$ or $E_{2}$).

**Fig. 8.**
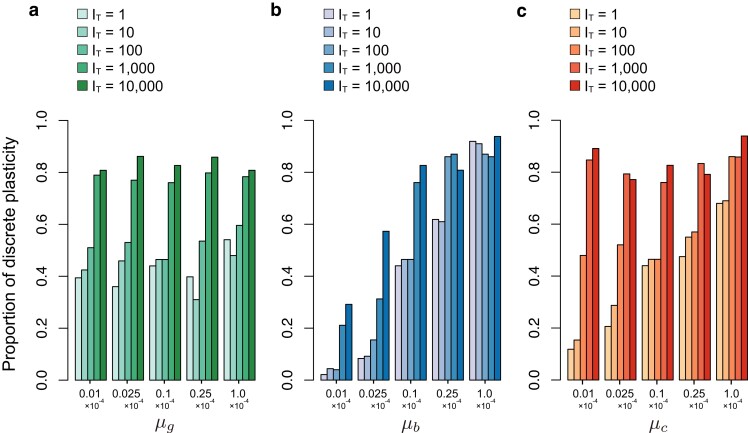
Proportion of simulation runs with a discrete phenotypic distribution. $\mu_{g}$ a), $\mu_{b}$ b), and $\mu_{c}$ c) were changed. Other parameters are the default values.

Based on this logic, we examined if the distribution of phenotype is discrete by using all successful runs in Fig. 2. We defined that intermediate phenotypes are those with both $w_{1}$ and $w_{2}$ less than 0.95. We checked the phenotypes of all individuals in the entire range of the environment and considered that the plasticity is discrete if intermediate phenotypes are less than 5%. Figure 8 shows the proportion of runs with a discrete phenotype distribution in each parameter set, where one of the three mutation rate parameters ($\mu_{g},\mu_{b},\mu_{c}$) was changed, while the other two were fixed. It was found that the proportion of discrete plasticity varies depending on the parameter sets. The proportion is large when $I_{T}$ is large, suggesting that the discrete plasticity is more likely to evolve when the network learn the two phenotypes one by one, rather than in a simultaneous manner. The proportion tends to be large when $\mu_{b}$ and $\mu_{c}$ are large. This pattern reflects that the well-evolved type-B and type-C genes are needed to make discrete plasticity so that the convergence to each equilibrium is sufficiently fast. The effect of $\mu_{g}$ is not well observed, consistent with the results in the previous section.

## Discussion

This study illustrates how discrete phenotypic plasticity could emerge through the evolutionary dynamics of a gene regulatory network. Our simulations in the full model with eight type-G genes showed that phenotypic plasticity could successfully evolve almost all parameter sets investigated (Fig. 2, Supplementary Fig. S4), indicating a marked ability of gene regulatory network to produce multiple phenotypes properly, but it takes a very long time. The simplified model with two type-G genes helped to understand the mechanism behind the acquisition of discrete plasticity (Figs. 3 and 4). (1) The most important aspect is the evolution of the phase portrait of the developmental process, which is orchestrated by the type-B genes. These genes must establish two stable equilibria, each corresponding to the optimal expression pattern under one of the two environments. This ensures that development can progress towards one of the two optima, effectively “memorizing” the adaptation to the environments within the gene regulatory network. (2) The type-C gene functions as a “switch” to enable the phenotype (expression) to cross the basin boundary during the developmental process. This dictates which optimum the development will proceed towards, effectively directing the phenotype without altering the type-B genes. These two factors represent the minimum conditions necessary for the evolution of discrete phenotypic plasticity through the evolution of a gene regulatory network. Additionally, for the phenotype distribution to become more discrete, the developmental process must occur rapidly enough for all individuals to reach their optimum phenotype. Failure to meet this condition may result in some individuals exhibiting intermediate phenotypes. This scenario is particularly relevant when intermediate environments are present, leading to a nondiscrete phenotype distribution along the environmental gradient (see Figs. 7 and 8). This reasoning is consistent with our simulations conducted under the full model with eight type-G genes.

There appear to be two primary pathways in the evolutionary process leading to the acquisition of plasticity, as illustrated in Figs. 5 and 6 (see also Figs. 3 and 4). When the environment changes frequently (i.e. small $I_{T}$, as shown in Fig. 5), the network initially evolves to produce a phenotype with maximum “average” fitness (i.e. $\sqrt{w_{1}w_{2}}$). Subsequently, both the ability to generate multiple phenotypes and the proper switching mechanism are acquired at a certain point in time. In such scenarios, the co-evolution of the type-B and type-C genes is crucial, and higher mutation rates at these genes accelerate the evolution of plasticity (Fig. 2). In contrast, when $I_{T}$ is large (Fig. 6), the network undergoes repeated adaptations to each environment and initially gains the ability to switch between the two phenotypes with only a few mutations (i.e. memorizing the two environments within a single network). Subsequently, the trigger for phenotype switching shifts from mutations to the sensor gene. Importantly, the network retains a robust memory capability, allowing it to remember the adaptive phenotype to past environments even after evolving for thousands of generations in different environments. In scenarios with slow environmental transitions, a high mutation rate at the type-C genes facilitates the evolution of plasticity by aiding in the acquisition of the proper switching mechanism. Conversely, a high mutation rate at the type-B genes slightly hinders the evolution of plasticity because a faster evolution of the network removes remnants of past adaptations, although it does promote the memory of the two optimum patterns (Fig. 2). Regardless of the specific conditions, we demonstrate that discrete phenotypic plasticity could eventually evolve across wide ranges of parameter sets, albeit requiring a significant amount of time, particularly in cases where $I_{T}$ is large.

Our study successfully demonstrated the evolution of discrete phenotypic plasticity, a feat that has proven challenging in previous models reliant on quantitative genetics, which tend to produce continuous distributions of phenotype (see the Introduction). Verbal arguments have suggested that considering the developmental process is crucial for understanding discrete plasticity, as the action of regulatory switches is necessary to establish such discrete traits (Schlichting and Pigliucci 1993; Via *et al.* 1995), yet mechanistic-based models of discrete plasticity have been relatively understudied. While some recent studies have explored the evolution of discrete plasticity (Svennungsen *et al.* 2011; Chevin and Lande 2013), these models have largely remained phenotype-based and have not provided insights into the underlying genetic mechanisms. Our theoretical framework offers a valuable tool for investigating the genetic properties involved in the evolution of discrete phenotypic plasticity, particularly in terms of gene expression, which is expected to differ both quantitatively and qualitatively from continuous plasticity.

This work demonstrates that discrete plasticity is more likely to emerge when mutation rates of the types-B and C genes are relatively high and environmental fluctuations are less frequent. Under these conditions, the convergence to stable equilibria in the developmental process tends to occur reasonably quickly, allowing the phenotype to reach one of the equilibria before adulthood even in intermediate environments, where the sensor gene may be only partially activated (see Fig. 8). This finding may shed light on the relatively rare empirical observations of discrete phenotypic plasticity, as environmental fluctuations in nature tend to occur over shorter time scales, such as seasonal changes. It is important to note that while our model assumes only two environments alternating with equal intervals, environmental changes in nature are much more complex. Given this complexity, the acquisition of discrete plasticity could be even more challenging in natural systems.

Our model considers a gene regulatory network, in which gene members regulate one another, that is, they behave as if they are transcription factors. In practice, transcription factors play the central role in development, and it is known that those genes likely self-regulate so that their expressions should be strongly enhanced or suppressed (Osborn *et al.* 2011; Bouldin *et al.* 2015). Provided this, our model would be reasonable in that we set the expression levels to be either 0 or 5 at the optimum states of $\vec{X}$. If we instead assumed intermediate optimum values (e.g. ${\vec{X}}_{1}=(1,1,4,4,4,4,1,1)$ and ${\vec{X}}_{2}=(1,1,4,4,1,1,4,4$), we found that $S_{p}$ was reduced and that the evolution of the discrete plasticity is less likely (see Supplementary Figs. S5, S6), suggesting that, in such situations, the evolution of the type-B genes that encompass fast convergence to equilibria in the developmental process is difficult.

In conclusion, our study illustrates that discrete phenotypic plasticity can evolve through the modulation of a regulatory network influenced by environmental cues sensed by a dedicated sensor gene. This modeling approach aligns well with empirical evidence indicating the involvement of hormones in shaping plastic traits during development. In many instances, environmental stimuli impact hormone secretion, while various hormone receptors, acting as transcription factors, regulate the developmental switch governing plastic traits based on hormone concentration levels during critical developmental periods (Nijhout 2003; Projecto-Garcia *et al.* 2017; Ledón-Rettig and Ragsdale 2021). This work underscores the significance of environmental cues in shaping gene regulatory networks, ultimately leading to the emergence of discrete phenotypic variations associated with developmental plasticity.

## Supplementary Material

jkae144_Supplementary_Data

## Data Availability

Codes used for simulations are available at https://github.com/TSakamoto-evo/plasticity. Supplemental material available at G3 online.
