# Supplementary material for: On the evolutionary origin of discrete phenotypic plasticity
Source: G3 (Bethesda). 2024 Jul 10;14(9):jkae144. doi: 10.1093/g3journal/jkae144 (PMC11373660; doi:10.1093/g3journal/jkae144)
Supplement: jkae144_Supplementary_Data [file jkae144_supplementary_data.pdf]

Supplementary materials for "On the Evolutionary Origin of Discrete Phenotypic Plasticity'

T. Sakamoto and H. Innan

April 22, 2024

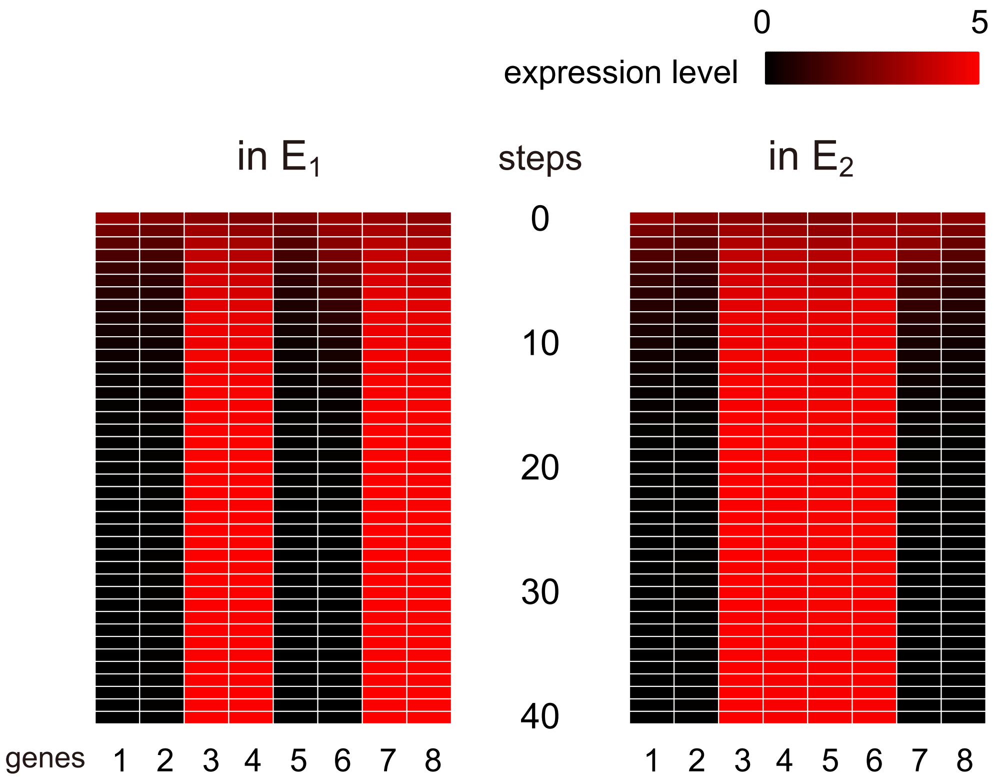

Figure S1: Changes of the expression levels of the n type-G genes through the development in the two environments, from embryo ($\tau = 0$) to adult ($\tau = \tau_l = 40$). The result shown is obtained at time point ▽T2 of Figure 5 in the main text.

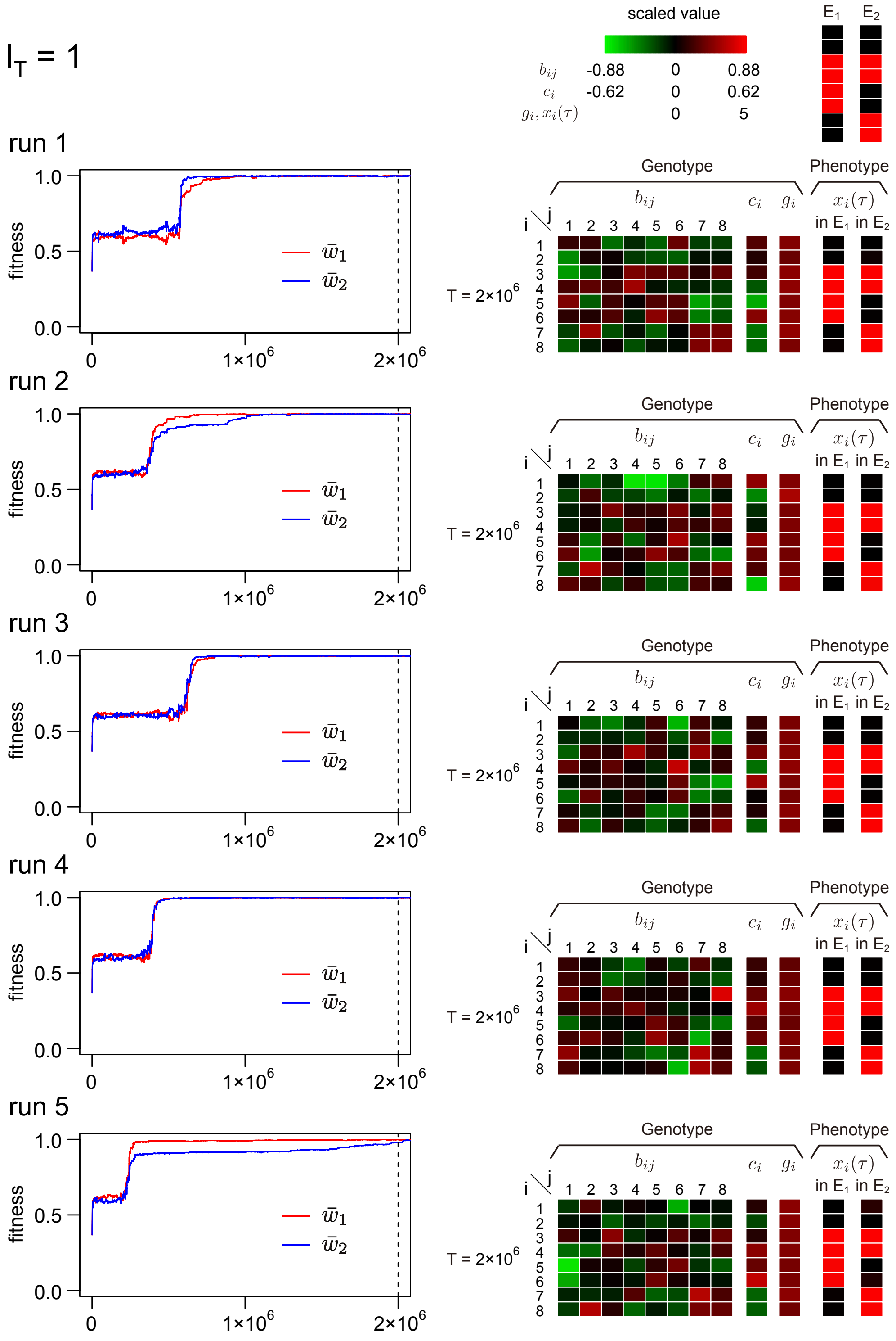

Figure S2: Five examples of a simulation run when $I_T = 1$. Parameters are the same as Fig. 5 in the main text. Fitness trajectory is shown on the left side, while genotype and phenotype at $T = 2 \times 10^6$ are shown on the right side.

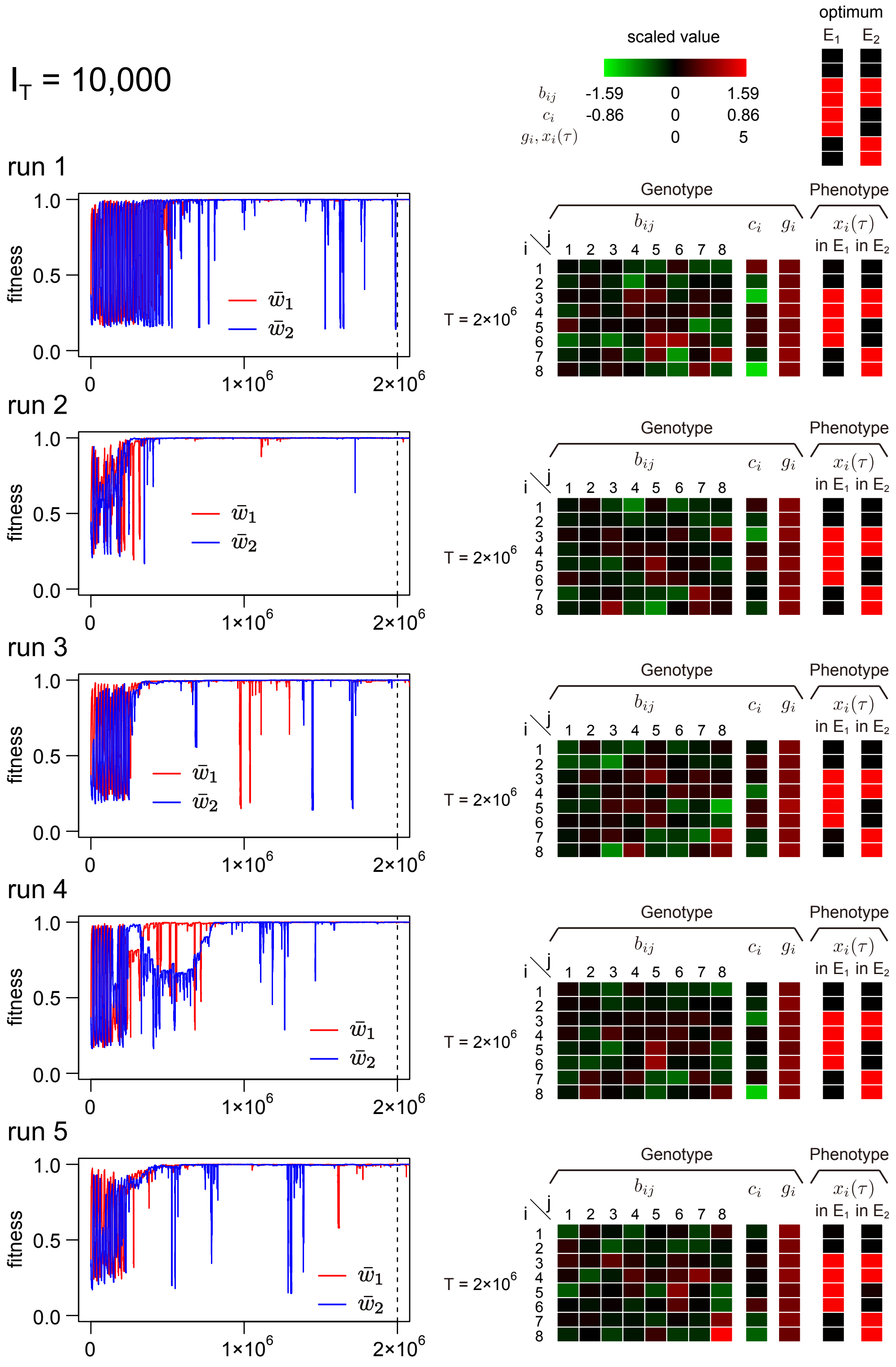

Figure S3: Five examples of a simulation run when $I_T = 10,000$. Parameters are the same as Fig. 6 in the main text. Fitness trajectory is shown on the left side, while genotype and phenotype at $T = 2 \times 10^6$ are shown on the right side.

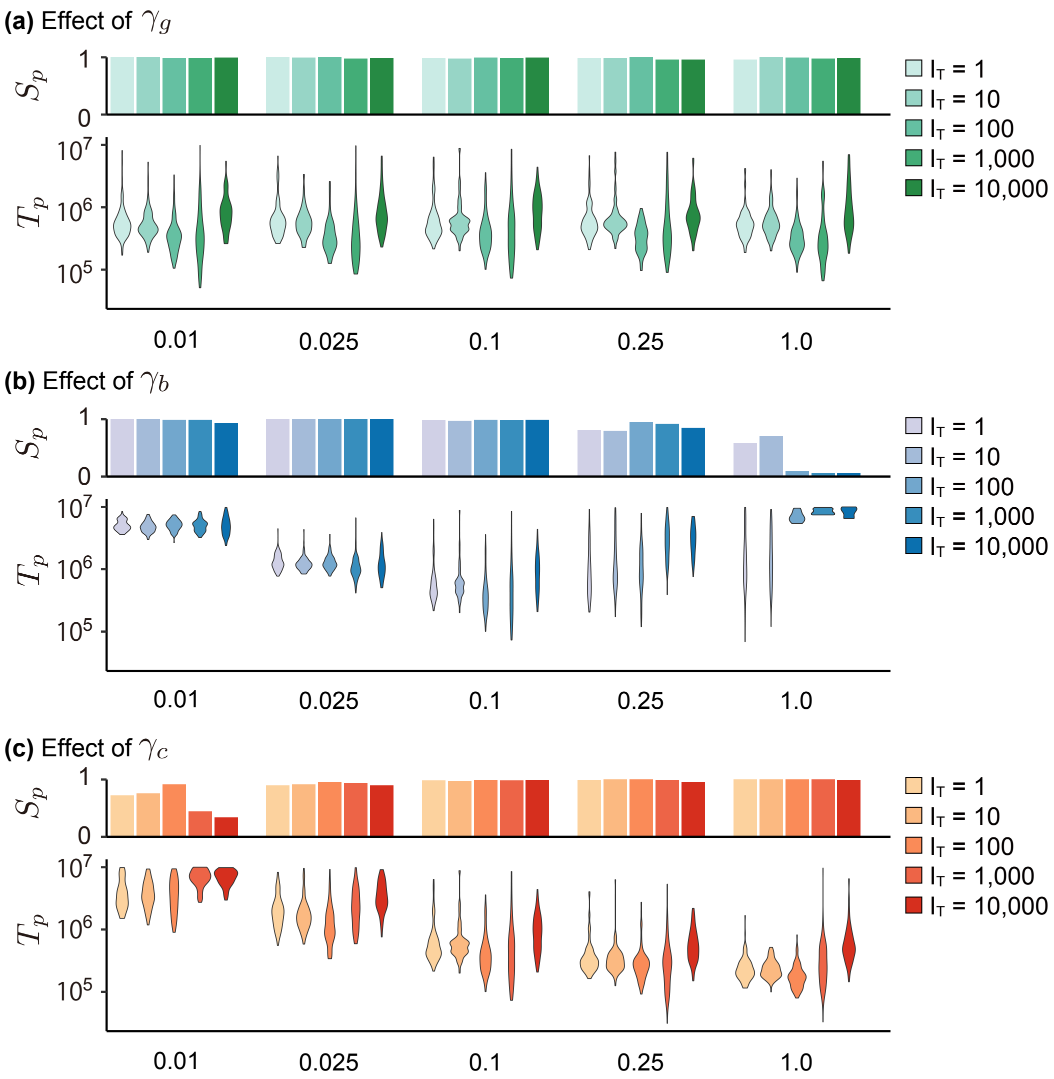

Figure S4: The effect of the mutational impact on the proportion of successful runs (S_p) and waiting time (T_p). The effect of γ_g (a), γ_b (b), and γ_c (c) are presented for various I_T. In each panel, S_p is shown in the upper bar plot while the distribution of T_p in successful runs is shown in the lower violin plot. For each γ_i, five γ_i values are examined. The other parameters are set to their default values (see the main text). The middle column ($\gamma_i = 0.1$) represents the results for the default parameters and is identical in (a), (b) and (c).

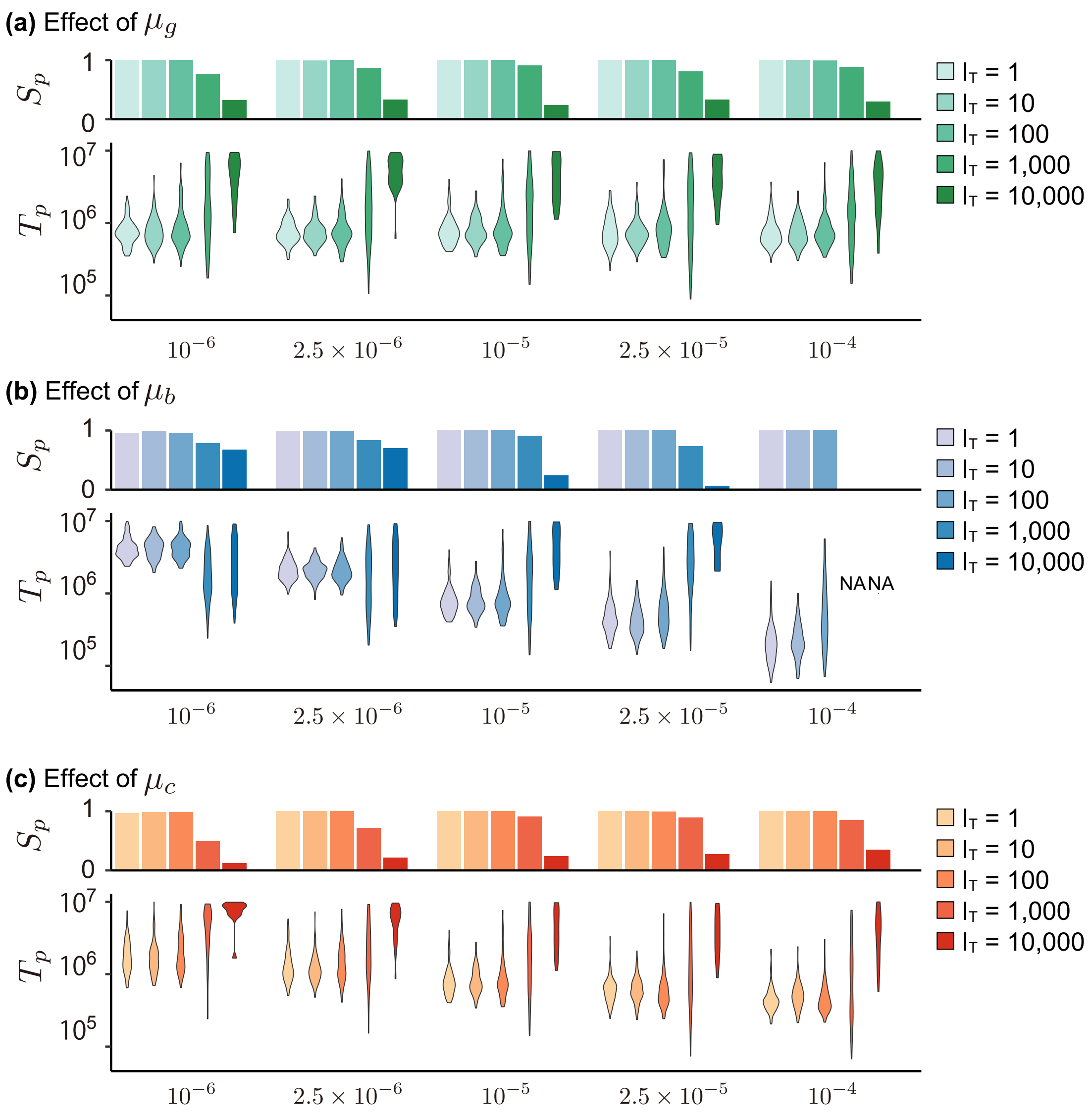

Figure S5: Evolution of plasticity when the optimal expression takes intermediate values. The effects of the three mutation rates ((a) μ_g, (b) μ_b, and (c) μ_c) are shown for various I_T. Setting is the same as Fig. 2 in the main text except for $\vec{X}_1 = (1, 1, 4, 4, 4, 4, 1, 1)$ and $\vec{X}_2 = (1, 1, 4, 4, 1, 1, 4, 4)$. The middle column ($\mu_i = 10^{-5}$) presents the results for the default parameters, so that the results are identical in (a), (b) and (c).

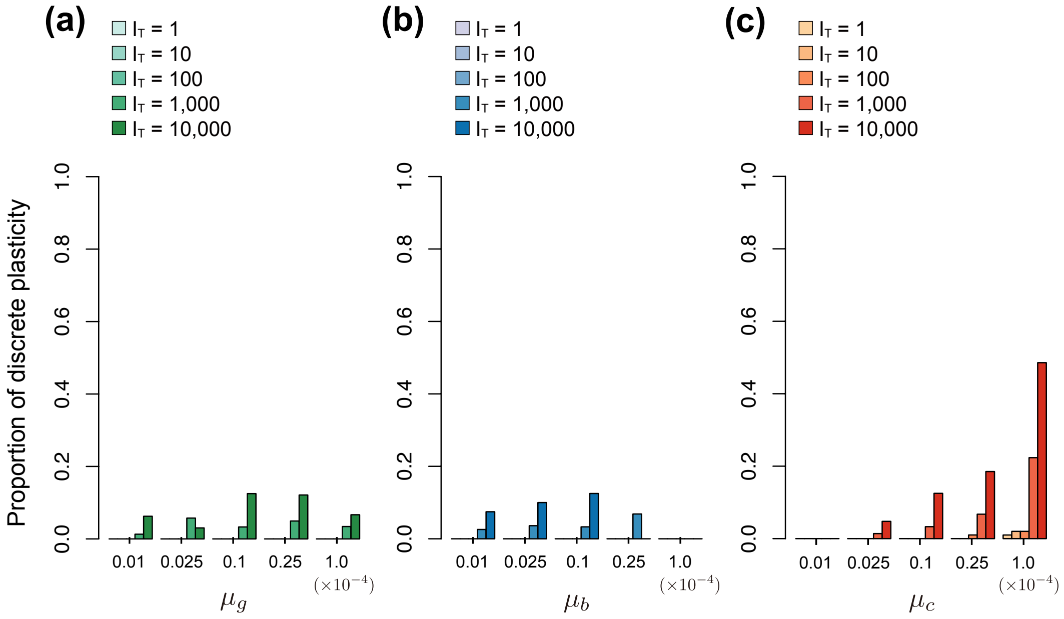

Figure S6: Discreteness of plasticity when the optimal expression takes intermediate values. Setting is the same as Fig. 8 in the main text except for $\vec{X}_1 = (1, 1, 4, 4, 4, 4, 1, 1)$ and $\vec{X}_2 = (1, 1, 4, 4, 1, 1, 4, 4)$.

1 Evolutionary dynamics when $I_T = 100$

In the main text, we focused on the two extreme cases with very small and very large I_T ($I_T = 1, 10^4$, respectively). We observed a mixed pattern of the two extreme cases with intermediate I_T. A typical pattern when $I_T = 100$ is shown in Figure S7. In the early phases, the mean fitness, $\bar{w}_1$, and $\bar{w}_2$, fluctuate around 0.6 (Figure S7). The range of fluctuation ($0.4 < \bar{w}_1, \bar{w}_2 < 0.8$) is larger than that in Figure 5, while smaller than that in Figure 6. Thus, we can see that this dynamics is somehow an intermediate pattern of the two extreme cases. In this stage, the type-G genes 1–4 are at their optimal expression levels, while the expression levels of the type-G genes 5–8 change as the environment changes (the middle panel in Figure S7b). After this phase, at $T \sim 1.8 \times 10^5$, the network acquires the ability of plasticity, and both $\bar{w}_1$ and $\bar{w}_2$ become ~ 1. Like the two extreme cases, b_{ij} and c_i play important roles in the development of plasticity (the bottom panel in Figure S7b).

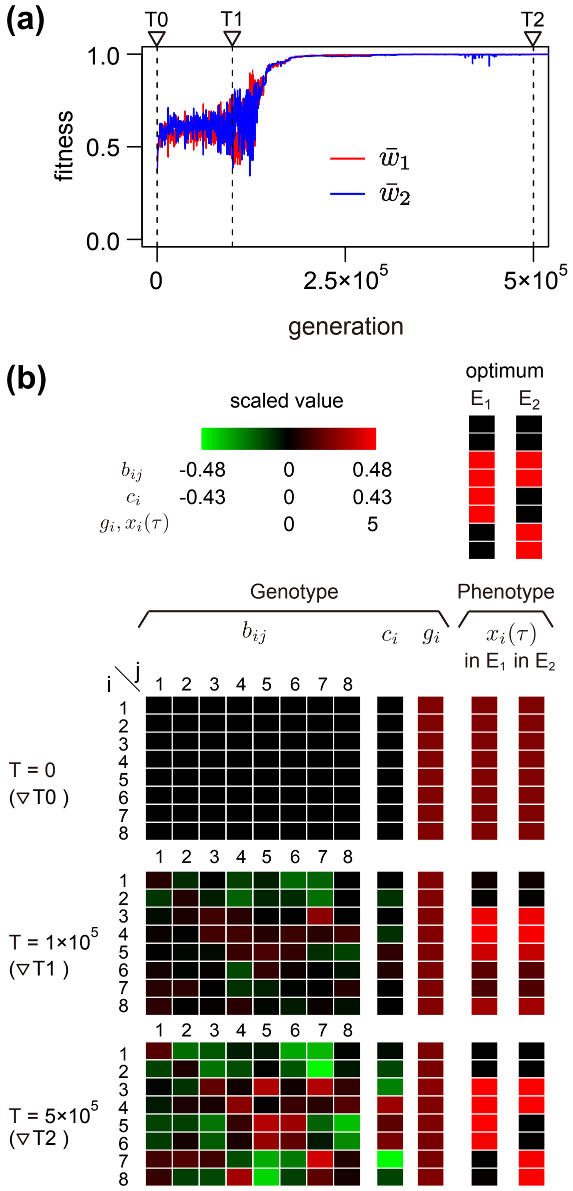

Figure S7: Evolution of phenotypic plasticity when $I_T = 100$. The result of a single representative run is illustrated. (a) Temporal change of fitness through evolution. The red and blue lines are the fitness in environment E_1 and that in E_2, respectively (i.e., $\bar{w}_1$ and $\bar{w}_2$). (b) Genotype and phenotype of an adult in each environment at three time points ($T = 0, 1 \times 10^5$, and 5×10^5) shown by the dashed vertical lines in (a). Phenotypes at the middle of the adult phase $\tau = 30$ are shown. Default parameter values were used (see Table 1 in the main text).
